# Characterisation of the Novel *Cutibacterium acnes* Phage KIT09 and First Report of CRISPR-Cas-Independent Bacteriophage Resistance in Phylotype IA1

**DOI:** 10.3390/ijms262412166

**Published:** 2025-12-18

**Authors:** Phuoc-Dung Nguyen, Koki Nakanishi, Huan Pham-Khanh Nguyen, Hoang Viet Nguyen, Masao Kitao, Masanao Yoshimoto, Kaeko Kamei

**Affiliations:** 1 Department of Functional Chemistry, Kyoto Institute of Technology, Kyoto 606-8585, Japan; dung.nguyenphuoc@gmail.com (P.-D.N.);; 2 College of Natural Sciences, Can Tho University, Campus II, 3/2 Street, Can Tho 94000, Vietnam; 3 Faculty of Medical Technology, Hanoi Medical University, Hanoi 100000, Vietnam; 4 Antimicrobial Technology Co., Ltd., Kyoto 601-8145, Japan

**Keywords:** *Cutibacterium acnes*, bacteriophage, resistant bacteria, CRISPR-Cas independent, pahexavirus

## Abstract

Despite being a commensal bacterium, *Cutibacterium acnes* has been widely considered a major opportunistic pathogen due to its capacity for biofilm production and inflammatory induction, causing device-related, post-implant infections, and skin inflammatory diseases. In this study, we isolated and characterised the novel bacteriophage *Cutibacterium acnes* phage KIT09 as a potential antimicrobial candidate for the treatment of *Cutibacterium acnes*-related infections such as acne vulgaris and postsurgical infections. Subsequently, phage-resistant bacterial mutants were generated through phage KIT09 exposure and characterised. Wastewater samples were collected for the isolation of *C. acnes* phages, followed by their characterisation using *C. acnes* National Institute of Technology and Evaluation (NITE) Biological Resources Center (NBRC) 107605 (phylotype IA1). Resistant mutants were isolated after prolonged exposure of the newly isolated phage to host bacteria and then characterised. A novel *C. acnes* phage, designated KIT09, was isolated, demonstrating prolonged bacteriolysis lasting up to 96 h at a multiplicity of infection of 10, and exhibiting high thermal and pH stability. Following sustained selective pressure by phage KIT09, three phage-resistant bacterial isolates were obtained, forming smaller colonies than the wild-type strain, but maintaining a high phage adsorption capacity (>90% after 20 min). Whole-genome sequencing revealed 12 nucleotide mutations across five genes, including six non-synonymous substitutions. Three genes encoding a two-component histidine kinase, DNA processing protein A (DprA), and a ThuA-containing domain protein were mutated in all resistant isolates. Characterisation of the novel phage KIT09 demonstrated its robust lytic activity and environmental stability against *C. acnes* phylotype IA1. Isolated resistant mutants retained high phage adsorption, accompanied by recurrent mutations in genes encoding a two-component histidine kinase, DprA, and a ThuA-domain protein, suggesting the presence of alternative, CRISPR-Cas–independent resistance mechanisms in *C. acnes*.

## 1. Introduction

*Cutibacterium acnes* (formerly named as *Propionibacterium acnes*) is a Gram-positive, facultatively anaerobic, rod-shaped bacterium that is highly prevalent in the human microbiota, especially in the pilosebaceous units of human skin [1]. Based on multilocus sequencing, *C. acnes* strains have been categorised into six phylotypes: IA1, IA2, IB, IC, II, and III [2]. Although considered commensal, overgrowth of a subset of *C. acnes* strains, particularly the acne-associated phylotype IA1, along with disruptions in the skin microbiome balance, increases pathogenicity by selecting more inflammation-inducing *C. acnes* strains, which can lead to acne vulgaris [3,4]. This was confirmed by an in vitro study showing that extracellular vesicles (EVs) produced by the phylotype IA1 strain from inflammatory acne lesions significantly increased the production of pro-inflammatory cytokines, thereby playing a crucial role in inducing inflammation in patients with acne vulgaris [5]. Furthermore, *C. acnes* is regarded as an opportunistic, invasive species that can be associated with deep tissue, postsurgical, or post-implant infections [6,7]. Critically, *C. acnes* can produce biofilms that protect it and other bacteria from antibiotics, aiding their adherence to sebaceous glands or prosthetic surfaces and thereby facilitating the onset of infection [8,9].

Currently, acne vulgaris is treated with topical and oral doxycycline or minocycline, whereas beta-lactam antibiotics or clindamycin are used to treat other related infections [10]. Additionally, a growing trend of resistance to broad-spectrum antibiotics such as clindamycin and clarithromycin has been observed in recent years [11,12]. Although the treatment of *C. acnes*-related infections varies by region, its high prevalence in the human microbiota could lead to exposure to antibiotics that are frequently misused, especially in developing countries. This raises concerns that new resistance patterns may emerge in the coming years. Consequently, research has shifted toward the development of bacteriophage-based treatments because of their specificity, bactericidal activity, and ability to penetrate biofilms. Significant efforts have been made to formulate phage cocktails against *C. acnes*, and the effectiveness of a single-phage formulation has been tested in animal models, which exhibited a reduction in *C. acnes*-induced inflammation [13]. Several phage resistance mechanisms of *C. acnes* have been identified, including the activation of CRISPR-Cas systems in phylotype II strains [14,15], restriction-modification (R-M) systems [16], and superinfection resistance following infection by pseudolysogenic phages [17,18]. However, the interactions between bacteria and other phylotypes remain unclear, although *C. acnes* phages exhibit limited genetic diversity, which in theory could make resistant strains more likely to emerge under selective pressure [19,20].

In this study, a new member of the *Pahexavirus* genus, designated *Pahexavirus* KIT09 (common name: *Cutibacterium* phage KIT09), was isolated and characterised using *C. acnes* National Institute of Technology and Evaluation (NITE) Biological Resources Center (NBRC) 107605 (phylotype IA1). In addition, the emergence of phage-resistant bacteria was first observed in a *C. acnes* strain lacking the CRISPR-Cas system. The identification and characterisation offered valuable insights into phage–host interactions and resistance development in *C. acnes*, emphasising the importance of comprehensive studies on phage-bacteria coevolution alongside application research.

## 2. Results

### 2.1. Isolation and Characterisation of Phage KIT09

#### 2.1.1. Morphology of Phage KIT09

**Figure 1:**
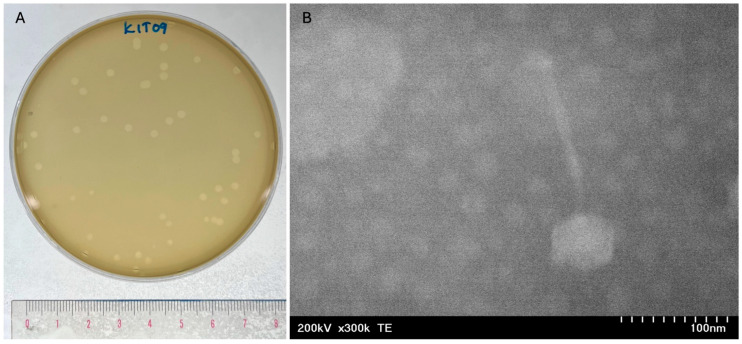
Plaque formation and morphology of phage KIT09. (**A**) Plaque formation of phage KIT09 on *C. acnes* NBRC 107605 bacterial lawns. Plaque formation was observed after 24 h of cultivation using the double agar overlay method. (**B**) Phage KIT09 morphology observed using an electron microscopy image at 300,000× magnification. The head diameter and tail length measurements were taken at a 100 nm scale using Fiji software.

Phage KIT09 was isolated from wastewater samples collected from various dental clinics in Hanoi, Vietnam, using *C. acnes* NBRC 107605 as a host. Phage KIT09 displayed a clear plaque with a size of approximately 2.39 ± 0.12 mm in diameter on *C. acnes* NBRC 107605 (Figure 1A). Negatively stained phage KIT09 virions were visualised using transmission electron microscopy (TEM) (Figure 1B). The structure of phage KIT09 exhibited a morphology with an isometric head with a diameter of 57.46 ± 0.95 nm and a non-contractile tail with a length of 148.49 ± 1.06 nm—previously described as the siphovirus morphology.

#### 2.1.2. Infectivity and Specificity of Phage KIT09

The host specificity of phage KIT09 was assessed by examining its infectivity against nine *C. acnes* strains belonging to phenotypes IA, IB, and II obtained from the NITE Biological Resource Center (NBRC) (Table 1). The spot assay of phage KIT09 at a titre of 10^8^ PFU/mL exhibited clear zones in six of the nine *C. acnes* strains, with clarity scores of 4 on Kutter’s five-point scale [21]. The efficiency of plating (EOP) was determined by proportionally calculating the average phage titre (plaque-forming units, PFU) of each test strain to that of the reference host (NBRC 107605). Only two strains (NBRC 113815 and 113816) were highly susceptible to phage KIT09 with EOP values > 0.5. Strains NBRC 113817 and 113819 exhibited moderate susceptibility, with values of approximately 0.35 and 0.36, respectively. The four remaining strains (i.e., NBRC 113818, 111530, 111595, and 113869) showed little to no susceptibility (EOP < 0.1), as indicated in Table 1.

#### 2.1.3. Infection Cycle and Stability of Phage KIT09

**Figure 2:**
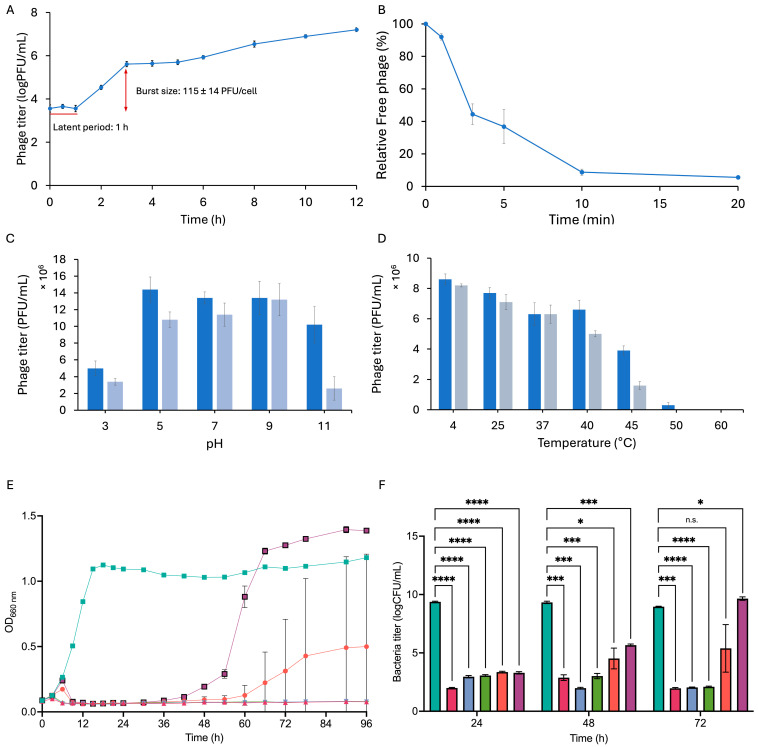
Properties of phage KIT09. (**A**) One-step growth describes the infection cycle of phage KIT09 to *C. acnes* NBRC 107605 (MOI = 0.0001). (**B**) Adsorption of phage KIT09 to *C. acnes* NBRC 107605 (MOI = 0.01). (**C**) pH stability of phage KIT09 was evaluated after 6 h (dark blue) and 24 h (light blue). (**D**) The temperature stability of phage KIT09 after 6 h (dark blue) and 12 h (grey blue) incubation. Bacteriolytic activity of phage KIT09 against host bacteria: (**E**) Phage KIT09 infected bacteria at different MOIs (0.001 to 10); (**F**) the bacterial titres. All experiments were performed in triplicate; data are presented as mean ± SD (n = 3). ****, *p* < 0.0001; ***, *p* < 0.001; *, *p* < 0.05; n.s., not significant. Control, no infection (teal); MOI = 10 (magenta); MOI = 1 (blue); MOI = 0.1 (green); MOI = 0.01 (salmon); MOI = 0.001 (purple).

The one-step growth curve experiment was conducted by infecting the host strain NBRC 107605 with phage KIT09 at a low multiplicity of infection (MOI) of 0.0001, ensuring a single infection of host bacteria. The one-step growth curve demonstrated a latent period of 1 h and a burst size of approximately 115 ± 14 PFU/cell (Figure 2A).

Titering of the free phage (non-adsorbed particles) over time was conducted to examine the adsorption of phage KIT09 on the host strain *C. acnes* NBRC 107605 (Figure 2B). Within the first two minutes, 52.88% of the phages were rapidly adsorbed by the bacteria. Subsequently, the adsorption rate decreased, with around 90% of the phages adsorbed after 20 min.

The stability of phage KIT09 with respect to pH and temperature was assessed by measuring its titre after exposure to various thermal and pH conditions. To examine pH stability, the phage was stored at pH 3–11 at 4 °C for 6 h and 24 h, whereafter phage titre was measured using *C. acnes* NBRC 107605 as the host (Figure 2C). The phage demonstrated significant stability across the examined pH range, with a titre decrease of less than 10-fold, even under the most acidic and basic conditions examined. Despite incubation for 24 h under the most acidic conditions (pH 3), the phage titre remained above 10^6^ PFU/mL. After 6 h of incubation under basic conditions (pH 11), the titre was approximately 10^7^ PFU/mL, and phage KIT09 retained activity slightly above 10^6^ PFU/mL after 24 h of incubation at pH 11.

Phage KIT09 was incubated at various temperatures, ranging from 4 to 60 °C, for 6 and 12 h, and then the phage titre was measured to analyse thermal stability (Figure 2D). The phage titre was retained at temperatures below 40 °C, and the phage lost infectivity at temperatures above 45 °C.

The bacteriolytic activity of phage KIT09 against *C. acnes* NBRC 107605 was evaluated by observing the bacterial growth after infection with phages at MOIs ranging from 0.001 to 10 (Figure 2E,F). At higher MOIs (0.1–10), phage KIT09 strictly inhibited the growth of *C. acnes* NBRC 107605 throughout the experiment (96 h), and kept the bacteria titre at approximately 10^2^ after 72 h (*p* = 0.0002, *p* < 0.0001, respectively). At low MOI values of 0.01 and 0.001, bacterial growth resumed at approximately 48 and 60 h, respectively. Additionally, in contrast to the rapid growth of the 0.001 MOI group from 48 h post-infection, the growth of bacteria infected with an MOI of 0.01 fluctuated. Thus, it resulted in the lower bacteria titre (logCFU/mL) of 5.388 (*p* = 0.2193) for MOI 0.01 versus 9.652 (*p* = 0.0359) for MOI = 0.001 at 72 h.

#### 2.1.4. Genomic and Phylogenetic Characterisation of Phage KIT09

Assembly of the phage KIT09 genome resulted in a single contig of 29,890 bp of double-stranded DNA (dsDNA), with an overall GC content of 54.02%. In total, 42 CDSs were identified, with 28 (66.67%) encoding functional proteins ranging from 97 to 922 amino acids (aa), whereas the remaining 14 (33.33%) were identified as putative proteins ranging from 63 to 189 aa (Table S1). The genome of phage KIT09 was submitted to GenBank (accession number: PX403247).

**Figure 3:**
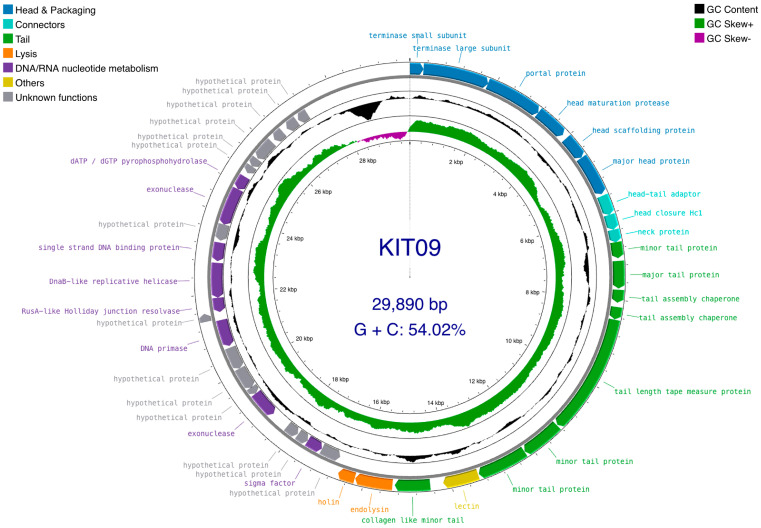
Phage KIT09 genome map. Circular dsDNA (outermost circle), including 42 ORFs organised into functional modules: head protein synthesis/assembly and DNA packaging (blue); head–tail connector proteins (cyan); tail protein synthesis/assembly (green); bacterial cell lysis (orange); DNA/RNA nucleotide metabolism (purple); other identified functions—containing lectin (dark yellow); and unknown function (grey). Arrowheads indicate the transcription direction. GC skew and GC plot are presented on the inner circle. The GC skew was calculated as (G−C)/(G+C), with GC+ (green) and GC− (purple). The GC plot (black) indicates GC% with values above average (pointed outward) and below average (pointed inward). The figure was generated using Proksee (accessed on 25 January 2025).

The 42 annotated CDSs were presented as functional modules, including head and DNA packaging, head–tail connectors, tail proteins, lysis enzymes, DNA/RNA nucleotide metabolism, and unknown functions (Figure 3). Specifically, the head assembly module consists of CDS_1 to CDS_6, which encode the terminase small/large subunits, portal proteins, head maturation proteases, head scaffolding proteins, and major head proteins. The next three CDSs (CDS_7–9) belonged to the connector module, with high similarity to the head–tail adaptor, head closure Hc1, and neck protein. The tail assembly module contained eight CDSs, including three copies of minor tail proteins (CDS_10, 15, and 16), a collagen-like minor tail protein (CDS_18), a copy of the major tail protein (CDS_11), a copy of the tail length tape measure protein (CDS_14), and two copies of tail assembly chaperones (CDS_12, 13). The two tail-assembly chaperones were connected by a ribosomal slippery sequence of (3′-GGGGAATAG-5′) located at position (8052..8060) near the stop codon of CDS-12. Most CDSs of known functional proteins were transcribed following the leading strand (clockwise direction), whereas the putative proteins and nucleotide metabolism-related enzymes were encoded on the lagging strand (anticlockwise) of the dsDNA genome. PhageTerm recognised 3′-cohesive overhang site (3′-COS) termini consisting of 11 bases (3′-TCGTACGGCTT-5′) at the genome end. Additionally, sequences encoding tRNAs, virulence factors, antibiotic resistance genes, transposons, or integrases were not identified using ARAGORN or ResFinder.

**Figure 4:**
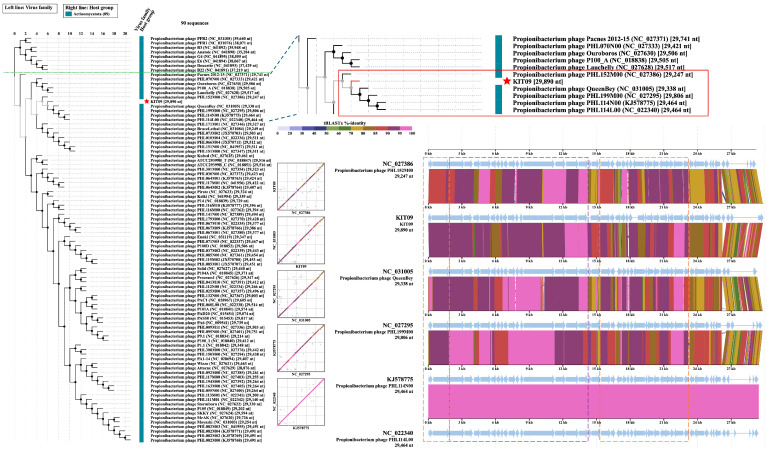
Species-level proteomic tree of phage KIT09 with a green dotted line dividing the two major clades. The zoomed-in minor clade containing phage KIT09 (red star) was presented in the red box. Sequence alignments of the phages within the same minor clade with phage KIT09 were generated using tBLASTx on the VipTree server; tBLASTx% identity is presented in a colour scale on the upper right of the alignment scheme. Structural proteins of the aligned phages were marked by a dashed purple box, and the enzymes related to important processes, such as DNA packaging, bacteriolysis, and DNA/RNA synthesis, were indicated by an orange dashed box.

Submission of the phage KIT09 genome to the Viral Proteomics Tree Server (VipTree), opting for prokaryotic host groups and the dsDNA nucleic acid type database (2230 sequences), placed phage KIT09 in the same clade as *Cutibacterium* phages (*Pahexavirus* genus). Thus, a subset of 90 *Cutibacterium* phage sequences was selected from the database to generate a species-level phylogenetic tree (Figure 4). The tree consists of two major clades, one of which includes eight *P. Freudenreichii* phages and the other contains 82 *C. acnes* phages. Subsequently, the genomes of phage KIT09 and its closest relatives (in the same minor clade), including *Cutibacterium* phage PHL152M00, QueenBey, PHL199M00, PHL114N00, and PHL114L00, were submitted to the Basic Local Alignment Search Tool for nucleotide sequences (BLASTn) and pairwise genome comparisons (JSpecicesWS Online Service) to examine query coverage, percent identity (Per. Ident), and average nucleotide identity using BLAST results (ANIb). All sequences exhibited identity values < 90% (Table 2), suggesting that phage KIT09 is a novel species belonging to the *Pahexavirus* genus (class *Caudoviricetes*).

Pairwise alignments using the VipTree server (translated Basic Local Alignment Search Tool for Nucleotide Sequences, tBLASTx) provided some insight into gene homology. Regarding the genes encoding structural proteins, such as the head, neck, and tail, phages in the same minor clade shared a high homology of 80–100% identity (Figure 4). Additionally, the head-to-tail adapter and head-to-tail stopper demonstrated lower homology (70–80%) to the neighbouring phages. In addition to structural proteins, enzymes crucial for phage DNA packaging or bacteriolysis, such as terminases, lysins, and holins, also share high homology. In contrast, most hypothetical proteins shared much lower homology, ranging from 50 to 80%. This trend was also observed in other phages within the same clade, except for PHL114N00 and PHL114L00, which had identical genomes.

### 2.2. Phage KIT09 Resistance Characterisation

#### 2.2.1. Generation of Phage-Resistant Bacterial Isolates During Coevolution with Phage KIT09

To generate phage-resistant bacteria, *C. acnes* NBRC 107605 was cultured overnight, mixed with phage KIT09 (MOI ≈ 1), and subsequently plated on soft agar, then overlayed with agar. After culturing at 37 °C for 20 days, eight colonies (R1–8) were collected from the double-layered agar and purified through four rounds of repetitive subculturing.

In the first round of purification, two types of spot assays were conducted to confirm the resistance of bacterial isolates to phages. Experiments R and P in Table 3 were performed to assess resistance against phage KIT09 and pseudolysogeny, respectively. In experiment R, phage KIT09 formed clear zones on the bacterial lawns of R1 and R7, suggesting that neither bacterium was resistant to phages. In experiment P, spotting R2, R3, and R4 on *C. acnes* NBRC 107605 bacterial lawns induced lysis, indicating that these three bacteria are pseudolysogens. Thus, we continued spot assays for R5, R6, and R8 in each round until the fourth round of repetitive purification, confirming that these three bacteria were resistant to phage KIT09 without pseudolysogeny. When the other four phages (KIT08 and KIT10–KIT12, previously isolated in our laboratory) were used instead of phage KIT09 to examine resistance (Experiment R in Table 3), the same results were obtained. This suggested that isolates R5, R6, and R8 were resistant to all available *C. acnes* phages without pseudolysogeny.

To ensure the absence of pseudolysogeny, DNA from isolates R5, R6, and R8 was extracted and subjected to PCR analysis. Electrophoresis of the PCR products showed no detectable bands corresponding to the DNA of the overhang sequence (LIU) [20] or the phage KIT09 major capsid protein (K09_mcp), suggesting a lack of phage KIT09 DNA as an episome (Figure S1). These results confirmed that the three isolates (R5, R6, and R8) were resistant strains with no pseudolysogeny.

#### 2.2.2. Characterisation of Phage-Resistant Isolates

**Figure 5:**
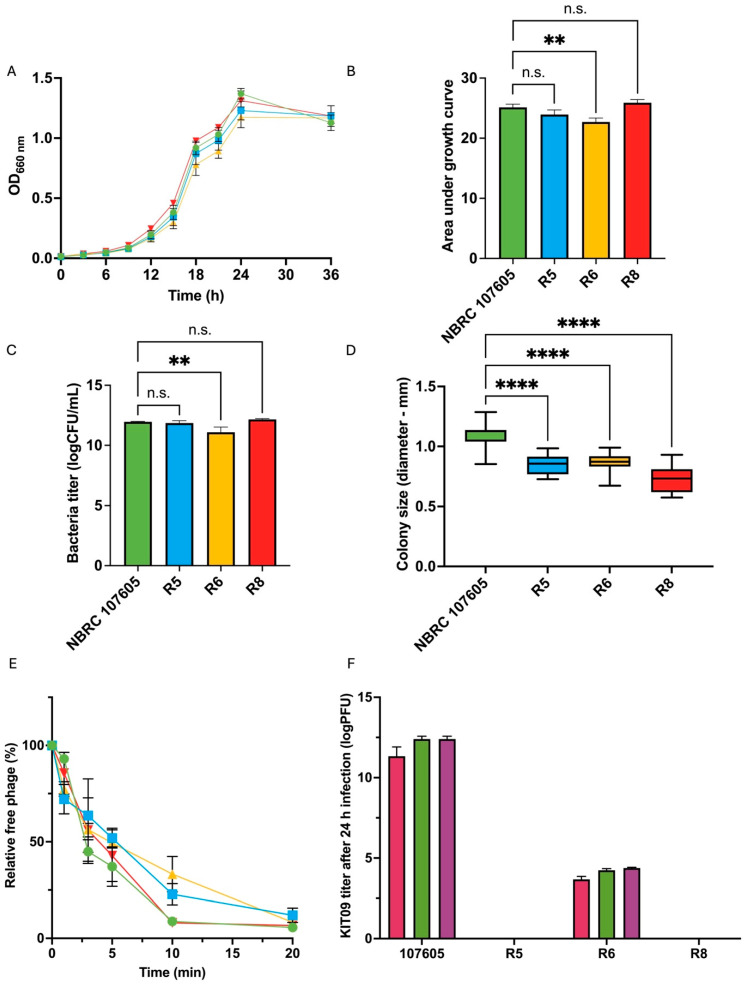
Characterisation of three resistant bacteria (R5, R6, and R8) and host strain (NBRC 107605): (**A**,**B**) represent the growth of the isolates in comparison with that of the host. (**A**) Growth curves of the host and resistant isolates, (**B**) the calculated AUC using Prism version 10, (**C**) the bacteria titre in log (CFU/mL after 24 h), and (**D**) the colony size (72 h). (**E**) Adsorption of phage KIT09 to the host (NBRC 107605) and resistant isolates. The host strain is illustrated in green, R5 in blue, R6 in yellow, and R8 in red. (**F**) The phage KIT09 titre was produced in the culture medium (24 h); MOI = 10 (magenta), MOI = 0.1 (green), MOI = 0.001 (purple). The experiments were performed in triplicate; data are presented as the mean ± standard deviation (SD). ****, *p* < 0.0001; **, *p* < 0.01; n.s., not significant.

Resistant isolates (R5, R6, and R8) were characterised and compared with the host strain (NBRC 107605) in terms of growth and phage interactions. The growth of the resistant bacteria and the host strain in liquid medium was monitored by measuring OD_660nm_ for 36 h and calculating the area under the growth curve (AUC) (Figure 5A,B). Resistant bacteria R5 and R8 demonstrated almost identical growth curves to that of the host strain, with no significant difference in AUC. Meanwhile, R6 exhibited slower growth than NBRC 107605, with significantly lower AUC values. Similarly, the bacterial titre in the 24-h culture of R6 was considerably lower than that of the host (Figure 5C). After plating the bacteria on GAM agar and culturing for 72 h under anaerobic conditions, all strains exhibited small, white, smooth, and slightly domed colonies, with sizes ranging from 0.73 to 1.09 mm. Despite morphological similarity, the resistant bacteria displayed significantly smaller colony sizes than the host (Figure 5D).

**Figure 6:**
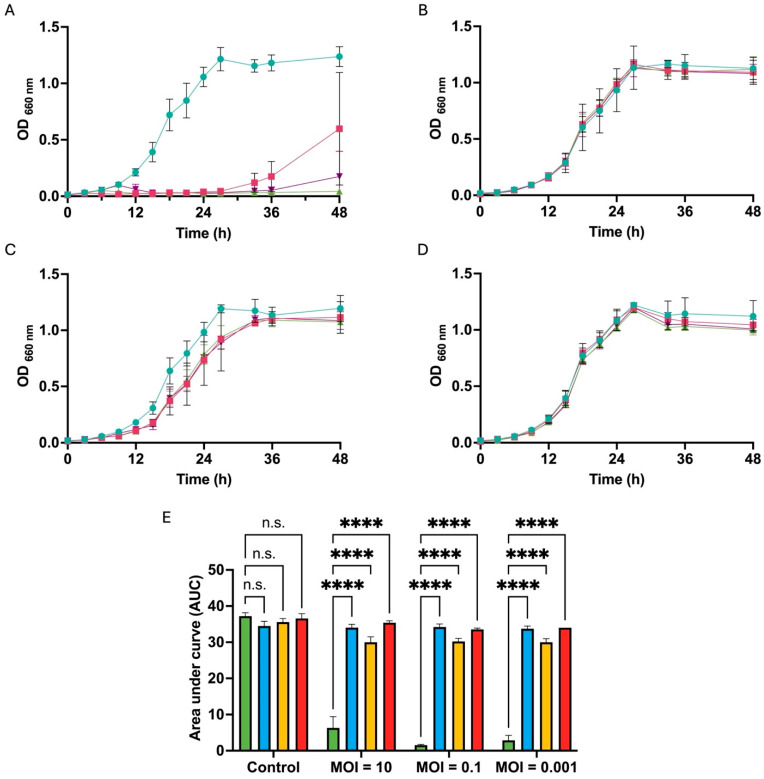
The growth kinetics of resistant bacteria in the presence of phage KIT09 at different MOIs. The growth curves of NBRC 107605 (**A**), R5 (**B**), R6 (**C**), R8 (**D**); 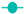, control (no infection); 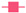, MOI = 10; 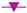, MOI = 0.1; 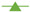, MOI = 0.001. (**E**) AUC after 48 h culture, calculated from growth curves by Prism software, NBRC 107605 (green), R5 (blue), R6 (yellow), R8 (red). The experiments were performed in triplicate; data are presented as the mean ± standard deviation (SD). ****, *p* < 0.0001; n.s., not significant.

The growth kinetics of resistant bacteria in the presence of different MOIs (10, 0.1, and 0.001) were monitored by measuring absorbance at 660 nm. Although phage KIT09 strictly inhibited the growth of the host bacteria (NBRC 107605), as shown in Figure 6A, the resistant bacteria R5 and R8 (Figure 6B,D) grew in a manner similar to that of the control (no infection), with no significant differences in the presence of different MOIs. Notably, for R6 (Figure 6C), the growth curve exhibited a slightly weaker growth with a reduced AUC in the presence of phage KIT09, especially at a higher MOI (MOI = 10). The AUC was also calculated to compare the growth inhibition exerted by phages on the resistant bacteria and the host (Figure 6E).

The adsorption and production of phage KIT09 by the resistant bacteria were examined to further observe the interactions between the resistant bacteria and phages. The results showed that the adsorption of phage KIT09 on all the resistant bacteria was almost the same as that on the original host strain, NBRC 107605 (Figure 5E). Among the resistant bacteria, phage KIT09’s adsorption to R8 exhibited the most similar pattern to that of the wild type (NBRC 107605), showing nearly identical adsorption rates during the first 10 min, with a relative free phage level below 10%. In contrast, slower adsorption was observed in R5 and R6, with relative free phage levels of 22.8% and 33.21%, respectively, at the same timestamp. However, similar to NBRC 107605, adsorption to all isolates was saturated after 20 min, with nearly 90–95% of the phage being adsorbed.

Notably, phage production was also observed in the supernatant of R6 infected with all three MOIs after 24 h of culture at 37 °C in anaerobic conditions, with a much lower titre than that of the NBRC 107605 strain (approximately 10^3^ compared to 10^12^ PFU/mL) (Figure 5F). Phage production was aligned with the slightly lower growth of R6 in the presence of phage KIT09 in the previous experiment, as shown in Figure 5A,B. This suggested that R6 was not entirely resistant to phage KIT09, whereas R5 and R8 developed complete resistance.

To analyse the mechanism of resistance acquisition against phage KIT09, the R5, R6, and R8 genomes were sequenced using the DNBSEQ platform for next-generation sequencing (NGS). Single-nucleotide polymorphisms (SNPs) or indels that differed from the wild-type *C. acnes* NBRC 107605 (GenBank accession: AP019723.1) were identified using Breseq version 0.39.0. In total, 12 different nucleotide mutations were identified in the resistant isolates, 9 in R5, and 8 each in R6 and R8 (Table 4). Among the 12 mutations, 7 were recorded in 5 genes: 1 synonymous mutation in *oxyR* and 6 non-synonymous mutations in *CacPP4_02910*, *sdhA_1*, *dprA*, and *CacPP4_19570*. Among the genes containing non-synonymous mutations, *sdhA_1* was found only in strain R5, whereas the other three genes, *CacPP4_02910*, *dprA*, and *CacPP4_19570,* were found in all three resistant bacterial genome sequences analysed. Notably, *CacPP4_02910*, which encodes a two-component histidine kinase, contained three mutations in each bacterial isolate. A network of associated proteins was constructed using STRING version 12.0 (Figure S2), suggesting that *CacPP4_02910* with other two-component histidine kinases (*CacPP4_10350* and *_08340*) and the hypothetical transmembrane protein CacPP4_02920 form a phosphorelay signal transduction system, which subsequently governs transcription regulation by seven DNA-binding response regulators (CacPP4_00510, 02900, 08920, 09120, 10360, 19410, and desR). The mutation H37R in dprA protein was identified in the SAM region, where many mutations were observed to disrupt the interaction of dprA-comE proteins. This interaction has been proven to be crucial in the competence shutoff observed in *Streptococcus pneumoniae* [22]. Additionally, the mutation S70W in *CacPP4_19570*, which encodes a ThuA domain-containing protein within the Class I glutamine amidotransferase-like family, was identified in the three resistant isolates.

Four mutations were identified in the intergenic sequences between *CacPP4_08380* and *_08390*, *CacPP4_12500* and *ocd*, *CacPP4_17970* and *_17980*, and *CacPP4_22410* and *_22420* in all sequenced strains. SAPPHIRE and BATTER were used to putatively locate the promoters and transcription terminations of these intergenic sequences to observe the potential impact of these mutations, which revealed that only the A > C mutation between *CacPP4_22410* and *22420* was located on a putative promoter. However, the *CacPP4_22420* CDS was encoded on the reverse strand, whereas the promoter was encoded on the forward strand.

## 3. Discussion

In this study, we identified and characterised a novel *Cutibacterium* phage KIT09 from dental clinic wastewater samples collected in Hanoi (Vietnam), using *C. acnes* NBRC 107605 as the host. Morphological and phylogenomic analyses placed phage KIT09 within the genus *Pahexavirus*, class *Caudoviricetes*. Phage KIT09 exhibits the characteristic features of this genus, including the previously described siphovirus morphology, with an isometric head (approximately 57 nm diameter) and a non-contractile tail (approximately 148 nm length), a double-stranded DNA genome of approximately 30 kb with moderate GC content (54.02%), and the presence of 3′-cohesive overhang termini for DNA packaging. Genomic comparison with closely related phages (PHL152M00, QueenBey, and PHL199M00) revealed 87–88% average nucleotide identity, suggesting that phage KIT09 is a novel species within this genus. Despite this genomic similarity, phage KIT09 exhibits distinct biological characteristics that distinguish it from other known pahexaviruses. Phage KIT09 exhibited bacteriolytic activity against six of the nine *C. acnes* NBRC strains, with extended inhibition of host bacterial growth, preventing bacteria from proliferating for up to 48 h at a low MOI of 0.001 and over 96 h at higher MOIs ranging from 0.1 to 10. This inhibition time was longer than that of previously isolated *C. acnes* phages with bacteriolytic activity, which was prolonged from 6 h to 72 h [23,24]. Notably, phage KIT09 showed MOI-dependent bacteriolysis; the higher the MOI, the longer the inhibition time. During infection, phage KIT09 underwent a short latent period of approximately 1 h, followed by the first burst, with a burst size of roughly 115 ± 14 PFU/cell, which was higher than some *C. acnes* phages with the same latent time, such as vB_CacS-HV1 with a burst size of 43 PFU/cell [23], and even longer latent time like phage PaP11-13 with a latent period of 5 h and burst size of 26 PFU/cell [25]. Despite having a similar genome size (approximately 29 kb), GC content, and high genomic homogeneity, the latent time and burst size of *C. acnes* phages varied greatly. The latent time ranged from 1 to 6 h, accompanied by burst sizes ranging from 26 PFU/cell, as in phage PaP11-13, to 2700 PFU/cell, as in phage 2012-15 [26]. These superior properties, including extended bacteriolytic activity and a moderate host range, indicate that phage KIT09 is a potential candidate for phage therapy against *C. acnes*-related infections. Additionally, phage KIT09 exhibited significant stability at physiological skin temperature (37 °C) and across acidic pH ranges typical of healthy skin, with less than a 10-fold titre decrease after 24-h incubation, making it suitable for incorporation into topical acne treatments and dermatological formulations.

Therapeutic activity against *acnes* has been achieved in vivo and in vitro by using phage-related products, including subcutaneous injection of phage suspension in *C. acnes*-infected mice [27], and recombinant endolysin derived from phage CAP 10-3, which exhibited a dose-dependent effect on *C. acnes* [28]. Despite numerous discoveries and studies exploring *C. acnes* phages as potential drug candidates, the interactions between phages and their hosts remain incompletely understood. The primary phage-resistant mechanism of *C. acnes* has been proposed to arise from the CRISPR-Cas system, which was found exclusively in type II strains [15]. Prior research by Marinelli et al. pointed out the correlation between matching spacers and phage resistance in CRISPR-Cas-type-I-E-harbouring *C. acnes* (phylotype II strains) [29]. Interestingly, the research of Liu et al. later demonstrated a weaker to no correlation between spacer and resistance [16]. Regarding the Restriction-Modification system, homology-based analyses have predicted three R-M systems in *C. acnes* clade IB, and only one in clade IA [16]. Additionally, Oxford Nanopore Technology (ONT) sequencing and Single Nucleotide Real Time (SMRT) methylome analysis only revealed significant methylation in single-locus sequence typing (SLST) H2 (of clade IB). Consistent with this finding, correlation with the R-M system has been illustrated in *C. acnes* KPA171202 (SLST H2—clade IB), with higher susceptibility against phage PAD20 in an R-M-deficient strain [16]. In contrast, no functional R-M system was detected in SLST A1 (clade IA1) or H1 (clade IB). Recent research has uncovered the pseudolysogenic cycle in several *C. acnes* phages, which are characterised by the presence of phage DNA in an episomal state inside the bacterial cell and spontaneous phage emanation [17,18,20]. Despite its instability, this state induces a superinfection-resistant state in *C. acnes*.

Importantly, these previously documented stable resistance mechanisms are yet to be identified in phylotype IA1 strains due to the absence of CRISPR-Cas and functional restriction-modification systems, while the only recorded resistance was in the pseudolysogenic state [17,18]. The question is whether phylotype IA1 strains lacking these conventional defence systems possess an alternative strategy to develop stable resistance to phage infection. In this study, resistant strains of *C. acnes* NBRC 107605 (type IA1) were isolated and characterised. Surviving colonies were isolated and purified through four subcultures, and screened to distinguish between superinfection resistance due to pseudolysogeny or intrinsic resistance. Furthermore, PCR analysis confirmed the absence of episomal phage DNA in resistant isolates. In comparison with the pseudolysogen induced by phage KIT08 (derived from the same host), the resistant strains demonstrated an almost identical growth rate to the wild-type, whereas the pseudolysogen displayed a markedly slower doubling time [17]. The isolates were also cultured with Mitomycin C (2 µg/mL) to assess prophage release into the bacterial culture and confirm the absence of lysogens (Figure S3). In alignment with the genomic sequence of phage KIT09 and its host, no signs of lysogeny were observed. Thus, isolates R5, R6, and R8 developed resistance to phage KIT09, representing the first resistant strain derived from a *C. acnes* strain without the CRISPR-Cas system. The resistant strains exhibited phenotypic characteristics distinct from those of the wild-type strain. All strains showed a considerably smaller colony size (23–33% reduction) than the host on agar medium. This may be attributed to trade-offs resulting from mutations that negatively affect the overall fitness of the bacteria. Similar reductions in colony size have been reported for phage-resistant variants of other species such as *K. pneumoniae* and *P. aeruginosa* [30,31]. Among the isolates, R5 and R8 demonstrated growth kinetics similar to those of the wild-type in liquid culture, whereas R6 exhibited significantly slower growth. This could be due to the mutation H37R in dprA protein, which may disrupt the interaction between dprA and comE protein complex. This interaction in *S. pneumoniae* has been reported to play a crucial role in the competence shutoff signalling system, without which the bacterial cells would remain in a constant competence state, depleting the cells’ nutrition and energy, and negatively impacting cell viability and physiology [22,32]. Another candidate is an intergenic mutation in the putative promoter near gene *CacPP4_22420*, encoding alanine racemase, which catalyses the conversion of L-alanine to D-alanine, which is critical for bacterial growth, as D-ala is a key component in the biosynthesis of cell wall peptidoglycans [33,34]. Furthermore, R6 also showed residual phage production (~10^3^ PFU/mL) when infected with phage KIT09, suggesting a partial resistance phenotype. This observation indicated that resistance mechanisms may vary in their completeness or efficiency, with R5 and R8 representing complete resistance, and R6 representing an intermediate phenotype.

Although spotting of *C. acnes* phages on the bacterial lawn of the resistant strain did not result in a lysis zone, liquid culture in the presence of phage KIT09 and R6 showed a slightly lower total area under the curve (AUC) than the control (without phage infection). Therefore, we examined the adsorption of phage KIT09 on resistant isolates. Intriguingly, all resistant isolates still adsorbed the phage at nearly the same rate as the wild-type, leaving only 5–10% of the free phage 20 min post-infection. This suggests that the bacteria developed resistance through a mechanism other than mutating the receptor to prevent adsorption, which agrees with the genetic information obtained from the short-read data of the resistant bacteria, as no mutation was found to occur directly in genes encoding wall teichoic acid synthesis or receptors [35]. Post-infection expression levels of phage genes encoding the major capsid protein (*mcp*) were determined by subjecting *C. acnes* NBRC 107605 and the resistant strain R5 total RNA samples extracted after 20 min infection of phage KIT09 to RT-qPCR (Figure S4). The expression levels of phage genes were significantly lower in the resistant strain than in the host. Thus, we hypothesised that mutations in resistant bacteria might downregulate the transcription of foreign DNA or protein synthesis in bacterial cells.

Additionally, nearly 40% (883 of 2302) of the CDSs in *C. acnes* NBRC 107605 encoded hypothetical proteins whose functions remain unknown, particularly sensor histidine kinases (HKs), which have been understudied. HKs are key enzymes in signal transduction under environmental stimuli, activating the response regulator in a phosphorylation network, leading to a cascade of cellular responses that have been reported to play a crucial role in numerous functions, such as cell viability, virulence, and antibiotic resistance [36,37]. Recent research has also shown that HKs participate in phage–host interactions, both directly and indirectly. For example, the two-component HK system PpoS/PpoR in *Marinomonas mediterranea* MMB-1 controls the CRISPR-Cas system in phage defence [38], and loss-of-function mutations in the HK RetS sensor induce phage resistance in *Pseudomonas aeruginosa* [39]. The phage-encoded PIT4 protein of *P. aeruginosa* phage LSL4 was found to interact with multiple HKs in *P. aeruginosa*, suggesting a role in the infection-exclusion mechanism [40]. In this study, all three phage-resistant *C. acnes*-derived strains had mutations in the same two-component histidine kinase (encoded by gene *CacPP4_02910*). While previous findings of the HK system in phage defence involved mediating CRISPR-Cas expression or interaction, mutations in the HK system in NBRC 107605 in the absence of CRISPR-Cas may represent a novel phage resistance mechanism in *C. acnes*.

In conclusion, the novel *Cutibacterium* phage KIT09, belonging to the genus *Pahexavirus*, demonstrated rapid infectivity and prolonged bacteriolytic activity against *C. acnes* NBRC 107605. Notably, this is the first report of resistant strains, derived from a *C. acnes* type IA1 strain lacking a CRISPR-Cas system. Characterisation and genetic sequencing of resistant bacteria have provided valuable insights into new issues that will be crucial for developing bacteriophages for future drug candidates. Our findings suggest that progress in phage discovery and formulation, coupled with comprehensive omics investigations, would deepen our understanding of phage–host dynamics before translating phage–host research into effective therapeutic strategies.

## 4. Materials and Methods

### 4.1. Materials

*C. acnes* strains used for phage characterisation (Table 1) were obtained from the Biological Resource Center of the National Institute of Technology and Evaluation (NITE), Tokyo, Japan. Wastewater samples used for phage screening and isolation were provided by dental clinics in Hanoi (Vietnam). AccuDia GAM medium (code: 05422) was obtained from SHIMADZU Corp. (Kyoto, Japan) for culturing the anaerobic bacteria. Agars BA-10 (agar plate, 1.5% *w*/*v*) and BA-30 (molten agar, 0.7% *w*/*v*) were obtained from Ina Food Industry Co., Ltd. (Nagano, Japan). The microbank was a product of IWAKI & Co., Ltd., Tokyo, Japan. Anaerobic cultivation was maintained using Jar/AnaeroPack-Anaero (Mitsubishi Gas Chemical, Tokyo, Japan).

### 4.2. Phage KIT09 Isolation and Characterisation

#### 4.2.1. Phage Enrichment and Isolation

Wastewater samples from dental clinics were centrifuged at 10,000× *g* for 10 min to remove particulates, and the supernatant was collected and used directly for enrichment with nine strains of *C. acnes* NBRC, following a previously described method, with some modifications [41]. Briefly, the sample supernatant (25 mL) was added to 25 mL of 2× GAM broth, inoculated with 0.2 mL of a 24-h culture of *C. acnes* strains (Table 1), and cultured under anaerobic conditions at 37 °C for 48 h. After incubation, the samples were centrifuged at 12,000× *g* and 4 °C for 10 min, and the supernatant was filtered through 0.20 µm filters (ADVANTEC, Tokyo, Japan). The filtered supernatant was precipitated using polyethylene glycol (PEG 6000, 4% *w*/*v*) and 0.5 M sodium chloride overnight at 4 °C, followed by centrifugation at 15,000× *g* for 90 min at 4 °C. The phage pellet was resuspended in SM buffer (200 mM NaCl_2_, 10 mM MgSO_4_, and 50 mM Tris-HCl, pH 7.5) and stored at 4 °C. The presence of the phages was examined using the double-layered agar method. Subsequently, the clear zone formed on the agar was collected in SM buffer, filter-sterilised, serially diluted, and mixed with a molten agar medium containing *C. acnes* NBRC 107605. The mixture was layered onto a GAM agar plate. Single plaques were selected and purified by repeating the double-layered agar method (plaque assay) at least three times [41]. Finally, *Cutibacterium* phage KIT09 was isolated.

#### 4.2.2. Morphology and Host Specificity

A high-titre phage KIT09 suspension (~10^10^–10^11^ PFU/mL) was dropped onto an elastic carbon support film on a copper grid (Ohken Shoji Co., Ltd., Tokyo, Japan) for 1 min to allow phage adsorption. The grids were stained with 1% (*w*/*v*) sodium phosphotungstate (pH 7.0) (Sigma-Aldrich, Burlington, MA, USA). Excess solvent was drained, and the grids were dried overnight in a desiccator chamber. Phage morphologies were observed under the transmission electron microscopy (TEM) mode of a scanning transmission electron microscope HD-2700 (Hitachi High-Tech Corp., Tokyo, Japan) at 200 kV [42]. The phage size was measured using Fiji version 2.16.0 [43].

Host specificity was determined by examining bacterial clearance on the bacterial lawn of nine different *C. acnes* NBRC strains, spotted with 5 µL drops of phage suspension (~10^7^ PFU/mL) in a spot assay. The clearance was ranked according to Kutter’s five-point scale (0–4) [21]. The efficiency of plating (EOP) was measured by spotting 10 µL of various 10-fold dilutions of phage KIT09 on different *C. acnes* NBRC strains and counting the formed plaques after overnight culture. EOP values were determined by calculating the percentage of plaques formed on each strain relative to the original host, NBRC 107605 [21]. The experiment was conducted in triplicate, and data were presented as mean ± SD.

#### 4.2.3. One-Step Growth Curve

A one-step growth curve experiment was performed to determine the latent time and burst size following previously described methods with slight modifications [26,44]. *C. acnes* NBRC 107605 was grown to the exponential phase (OD_660nm_~0.5), inoculated with phages at a multiplicity of infection (MOI) of 0.0001, and stored at 4 °C/60 min for pre-adsorption. Subsequently, the mixture was centrifuged at 12,000× *g* and 4 °C for 5 min, and the pellet was suspended in 10 mL of fresh GAM liquid medium and incubated at 37 °C under anaerobic conditions. Subsequently, 100 µL of the culture was collected at 0.5- and 1-h intervals for 6 h and at 2-h intervals for 12 h. The phage titre in the collected culture was quantified using a plaque assay by mixing fresh bacterial suspension (OD_660nm_~0.5) and GAM molten agar (0.7% *w*/*v*), and then layering on GAM agar (1.5% *m*/*v*). Clear plaques were then counted to calculate the PFU. The experiment was conducted in triplicate, and the data were presented as mean ± SD.

#### 4.2.4. Adsorption Assay

Phage adsorption was performed according to a previously reported method, with some modifications [26,45]. *C. acnes* NBRC 107605 was grown to the exponential phase (OD_660nm_~0.5), inoculated with phages at an MOI of 0.01, and incubated at 37 °C. At 0 (before infection), and at 1-, 3-, 10-, and 20-min post-infection, 1 mL of culture medium was collected using a syringe and immediately filtered through a 0.20 µm filter (ADVANTEC, Tokyo, Japan) to collect free phages. The plaque-forming units (PFU) of free phages were determined by a plaque assay using the double-layered agar method with *C. acnes* NBRC 107605. The adsorption rate and relative percentage of free phages were calculated using the following formula, with the titre at 0 min used as the control. Adsorption of phage KIT09 onto resistant bacteria was conducted similarly; however, the original host was replaced with resistant isolates (R5, R6, and R8) for the adsorption target. The experiments were performed in triplicate, and the data are presented as mean ± SD. The adsorption rate, measuring the percentage of phage absorbed, was calculated using the following equation:

$$adsorption\;rate=\frac{\left(control\;titer-residual\;titer\right)}{\left(control\;titer\right)}\times 100$$

where the control titre is the phage titre (PFU/mL) at time 0 (before infection), and the residual titre is the phage titre (PFU/mL) in the culture medium at each time point. The percentage of free phages was determined as:

$$free\;phage\left(\%\right)=100-adsorption\;rate\left(\%\right)$$

#### 4.2.5. Phage Stability

For pH stability, phage KIT09 (~10^7^ PFU/mL) was incubated in SM buffer adjusted to pH 3, 5, 7, 9, and 11 with NaOH or HCl, and the titre was examined at 6 and 24 h post-incubation [46]. In terms of thermal stability, phage KIT09 (~10^7^ PFU/mL) was incubated at 4, 20, 37, 40, 45, 50, and 60 °C, and the titre was analysed at 6 and 12 h post-incubation [46]. Titres were measured using the double-layered agar method. All experiments were performed in triplicate, and the data were presented as mean ± SD.

#### 4.2.6. Bacteriolytic Activity

Bacterial growth was measured after infection with phages at different MOIs to investigate bacteriolytic activity. Briefly, 500 µL of *C. acnes* NBRC 107605 (OD_660nm_~0.5) was mixed with 500 µL of adjusted dilutions of phage KIT09 to produce MOIs ≈ 0.001, 0.01, 0.1, 1, and 10, respectively. The mixtures were transferred to 5 mL of fresh GAM medium and cultured statically at 37 °C under anaerobic conditions. For the control, 100 µL of SM buffer was used instead of the phage solution. The mixtures were agitated by vortexing to ensure uniform distribution before being monitored by measuring the OD_660nm_ at intervals of 3 h until 96 h. At 24, 48, and 96 h, 100 µL was collected from each mixture, submitted to 10-fold serial dilutions, and spread on GAM agar plates for bacteria counting (CFU). The experiment was conducted in triplicate, and the data are presented as mean ± SD.

#### 4.2.7. Genome Analysis

Genomic DNA was extracted from 900 µL of filtered phage KIT09 lysate (10^11^ PFU/mL) using the QIAGEN DNeasy Blood & Tissue Kit (QIAGEN, Hilden, Germany), with bacterial DNA/RNA removal before the extraction using Recombinant DNase I and RNase A (Takara Bio Co., Ltd., Shiga, Japan), based on a previously described method [47]. BGI Japan performed genome sequencing using a DNBseq platform with paired-end reads of 150 bp. Raw data were subjected to Fastp version 0.22.0 to detect/trim adapters (read 1: AAGTCGGAGGCCAAGCGGTCTTAGGAAGACAA; read 2: AAGTCGGATCGTAGC CATGTCGTTCTGTGAGCCAAGGAGTTG) to filter low-quality reads and contaminants [48]. The reads before and after trimming were qualified using FastQC [49]. Genome assembly was performed by Shovill pipeline version 1.0.9 (https://github.com/tseemann/shovill, accessed on 6 November 2024), utilising seqtk version 1.4 (https://github.com/lh3/seqtk, accessed on 6 November 2024) for collecting raw reads statistics & subsampling reads to the ideal depth of 100×; correcting reads by Lighter version 1.1.3 [50], overlapping/stitching paired-end reads by FLASH version 1.2.11 [51], Velvet version 1.2.10 as genome assembler [52]; read mapping by BWA and SAMtools for assembly polishing using Pilon version 1.24 [53]. The assembly was examined using QUAST version 5.0.0 [54] for contig continuity, assembly size, and N50. checkV version 1.0.3 [55] was used to assess contig quality, host genome contamination, and completeness. Read mapping back to the assembly, extraction of unmapped reads, and remapping against the host genome to check for contamination were carried out using BWA-MEM2 version 2.2.1 [56] and SAMtools version 1.9 [57]. Genomic annotation was performed using Pharokka version 1.7.0 [58], with gene prediction using Pyrodigal-gv version 0.3.2 [59], followed by functional annotation through comparison with the PHROGs database [60]. Hypothetical protein analysis was performed by piping the results from the Pharokka to the Phold pipeline (https://github.com/gbouras13/phold, accessed on 25 November 2024) to structurally present proteins using the ProstT5 protein language model and comparing them against the modelled protein structure in FoldSeek [61,62]. Manual curation of the resulting annotations was conducted using the NCBI Blast Suite (https://blast.ncbi.nlm.nih.gov/Blast.cgi, accessed on 10 December 2024) [63], HHPred (against PDB, UniProt-SwissProt-viral, NCBI_Conserved_Domains database) [64]. The Pharokka pipeline also compared the predicted genes against databases of antimicrobial resistance genes (CARD) [65], virulence factors (VFDB) [66], CRISPR [67], and tRNA via ARAGORN [68] to identify these factors in the phage genome. PhageTerm version 4.2b0 was used to detect the phage termini and packaging mechanisms [69]. PRFect version 0.41 predicted ribosomal frameshifts in viral genomes [70]. The annotated genome of phage KIT09 was deposited in GenBank (accession number: PX403247).

The genome map of phage KIT09 was generated using the Proksee server (https://proksee.ca/, accessed on 25 January 2025). Proteomic-based phylogenetic analyses were conducted by submitting the phage KIT09 genome to the VipTree webserver (https://www.genome.jp/viptree/, accessed on 30 January 2025). Therefore, a subset of *Cutibacterium* phages (formerly called *Propionibacterium* phages) was selected to construct a species-level phylogenetic tree.

#### 4.2.8. Phage-Resistance Induction

Phage-resistant bacteria were isolated and distinguished from pseudolysogen following previously mentioned methods, with some modifications [17,18,30,71]. The overnight culture of *C. acnes* NBRC 107605 was mixed with phage KIT09 (MOI ≈ 1) and GAM soft agar (0.7% *m*/*v*), and then layered on GAM agar (1.5% *m*/*v*). The plates were cultured at 37 °C for 20 days under anaerobic conditions, and colonies of *C. acnes* NBRC 107605 were confirmed. Eight colonies (R1–R8) were randomly selected and passaged four times on a new GAM agar plate to purify the isolate and remove the phages. For each subculture, colonies were collected and stored in a microbank at −80 °C.

Additionally, two types of spot assays were carried out at each round of purification of isolates: spotting the *C. acnes* phages (KIT08–KIT12, including KIT09, isolated in our laboratory) on the isolate’s bacterial lawn to check for resistance to phages, and spotting bacterial isolates on the host (NBRC 107605) bacterial lawn to check whether bacterial isolates are pseudolysogens which induce the lysis of host bacteria. In both assays, the agar plates were incubated at 37 °C for 24 h under anaerobic conditions, and plaque formation was observed. To examine whether phage KIT09 was in a prophage state, 100 µL of the overnight culture of isolates was transferred to 10 mL fresh GAM medium with Mitomycin 2 µg/mL and cultured for 12 h at 37 °C. The supernatant was collected and filtered through a 0.2-µm filter, and spotted on an NBRC 107605 bacterial lawn to check for the presence of prophage by observing bacteriolysis.

In the fourth passage, resistant isolate colonies were collected, and DNA was extracted using QIAGEN DNeasy Blood & Tissue Kit (Germany). The DNA samples were subjected to PCR amplification of the overhang sequence (LIU) using primers reported previously [20] and the phage KIT09 major capsid protein (K09_mcp) to examine the presence of the episomal phage KIT09 genome, as in the pseudolysogen. KOD One PCR Master Mix was used with primers (Table S2) and amplified with 35 two-step heat cycles: first step of denaturation (98 °C/10 s) and second step of annealing/elongation (68 °C/5 s). Phage KIT09 DNA was used as the positive control, host DNA (NBRC 107605) was used as the negative control, and nuclease-free water was used as the no-template control. All amplicons were electrophoresed on 3% agarose gel at 100 V for 40 min.

#### 4.2.9. Phage-Resistance Characterisation

Phage-resistant isolates were characterised with regard to biological properties such as growth, colony size, phage adsorption, and inhibition following previously mentioned methods, with some modifications [30,31]. The growth of phage-resistant bacteria was characterised by measuring the OD at 660 nm at 3-h intervals for 24 h. Briefly, 100 µL of bacterial culture was collected, serially diluted, and spread on GAM agar to count colonies at 12 h and 24 h to quantify the actual bacterial growth. Experiments to determine phage KIT09 adsorption to resistant bacteria were conducted as described for the adsorption assay. All experiments were performed in triplicate, and the data are presented as mean ± SD.

Phage dynamic interaction with resistant bacteria was carried out by infecting 100 µL bacteria culture (OD_660nm_~0.5) of the resistant isolates (R5, R6, and R8) and NBRC 107605 with 100 µL of phage KIT09 at MOI = 10, 0.1, and 0.001 in 5 mL GAM. SM buffer rather than phage suspension was used as a negative control. The growth of bacteria in the presence of phages was monitored by measuring OD_660nm_ at 3-h intervals for 48 h. The area under the curve (AUC) was calculated using Prism software. At 24 h, 100 µL of each culture was collected, serially diluted in SM buffer, and spotted onto NBRC 107605 bacterial lawns for counting clear plaques to determine PFU. All experiments were performed in triplicate, and the data are presented as mean ± SD.

The extracted DNA from phage-resistant isolates R5, R6, and R8 was subjected to genome sequencing on the DNBseq platform by BGI Japan using 150-bp pair-end reads. Raw data were processed using Fastp version 0.22.0 for adapter trimming and quality filtering. Clean reads were aligned to the NBRC 107605 reference genome (GenBank accession: AP019723.1) to identify mutations using Breseq version 0.39.0 [72]. NCBI BLASTp, HHPRED, InterProScan, and STRING version 12.0 were used to obtain information about the hypothetical proteins with identified mutations [73,74]. SAPPHIRE and BATTER were used to identify promoters and transcription terminations in intergenic sequences with mutations [75,76].

Resistant bacteria and the host (NBRC 107605) were infected with phage KIT09 at MOI = 1 for 20 min, followed by a wash and resuspension of the pellet in PBS, before being submitted to cell wall disruption using glass beads (0.1–0.15 mm) and RNA extraction using the ISOGEN (Nippon Gene Co., Ltd., Tokyo, Japan) kit. The extracted total mRNA was converted to cDNA using ReverTra Ace qPCR RT Master Mix with gDNA Remover (TOYOBO Co., Ltd., Osaka, Japan). The expression level of the phage gene encoding the major capsid protein (mcp) and NBRC 107605 16S was examined via real-time qPCR using TB Green Premix Ex Taq II (Tli RNaseH Plus) (Takara Bio Co., Ltd., Shiga, Japan). The primers used for qPCR are listed in Table S2. All experiments were performed in triplicate, and the data are presented as the mean ± SD.

## Figures and Tables

**Table 1:** Host specificity of phage KIT09.

NBRC No.	Phylotypes * (MLST **)	Source of Isolation *	Clarity Score ^†^	EOP
107605	IA1	Facial acne	4	1.00 ± 0.18
113815	IA2	Human skin (glabella) of a 30-year-old female	4	0.63 ± 0.04
113816	IA2	Human skin (back) of a 40-year-old male	4	1.25 ± 0.03
113817	IB	Human skin (cheek) of a 30-year-old female	4	0.35 ± 0.07
113818	IB	Human skin (chin) of a 40-year-old male	0	0
113819	II	Human skin (cheek) of a 40-year-old male	4	0.36 ± 0.07
111530	IB	Facial acne of a 35-year-old man	0	0
113595	IB	Human skin	0	0
113869	IA2	Human skin (nasal area) of a 30-year-old female	4	0.01 ± 0.004

* Based on NBRC database. ** MLST, multi-locus sequence typing. ^†^ Spot assay was performed using a phage titre of 10^8^ PFU/mL. The clarity score was ranked according to Kutter’s scale: 4, clear plaques/zone (complete lysis); 3, clearing with faintly hazy background; 2, substantial turbidity; 1, some individual plaques; 0, no clearing. EOP was presented as average ± standard deviation.

**Table 2:** Alignment parameters of phage KIT09 to its five other phages within the same minor clade.

Phage Name (Accession)	Genome Size (nt)	Query Cover	E-Value	Per. Ident	ANIb
*Propionibacterium* phage PHL152M00 (NC_027386)	29,247	98%	0	89.25%	87.58%
*Propionibacterium * phage QueenBey (NC_031005)	29,338	98%	0	88.82%	88.23%
*Propionibacterium * phage PHL199M00 (NC_027295)	29,806	100%	0	88.31%	87.65%
*Propionibacterium* phage PHL114N00 (KJ578775)	29,464	98%	0	88.91%	87.99%
*Propionibacterium* phage PHL114L00 (NC_022340)	29,464	98%	0	88.91%	88.00%

**Table 3:** Resistance properties of bacterial isolates in four subcultures for purification.

Subculture (Passage)	R1		R2		R3		R4		R5		R6		R7		R8	
Experiment *	R	P	R	P	R	P	R	P	R	P	R	P	R	P	R	P
1st	◯	✕	✕	◯	✕	◯	✕	◯	✕	✕	✕	✕	◯	✕	✕	✕
2nd	–	–	–	–	–	–	–	–	✕	✕	✕	✕	–	–	✕	✕
3rd	–	–	–	–	–	–	–	–	✕	✕	✕	✕	–	–	✕	✕
4th	–	–	–	–	–	–	–	–	✕	✕	✕	✕	–	–	✕	✕
Properties																

* Experiment R: Phage KIT09 was spotted on each bacterial isolate lawn to examine resistance against phage KIT09. Experiment P: each isolate was spotted on the lawn of the host bacterium (NBRC 107605) to assess pseudolysogeny. ◯: form clear zone; ✕: turbid zone; –, not measured. 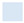 No resistance; 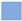 Pseudolysogeny-induced resistance; 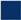 Resistance without pseudolysogeny.

**Table 4:** SNPs identified from NGS data of resistant strains R5, R6, and R8 using Breseq version 0.39.0.

Position	Mutation	R5	R6	R8	Annotation	Gene	Description (BlastP, HHPRED, InterPro)
339,446	C→T			√	G298D (GGC→GAC)	*CacPP4_02910* ←	two-component sensor Histidine kinase
339,465	C→G	√			V292L (GTC→CTC)		
339,726	G→T		√		L205I (CTC→ATC)		
924,585	G→A	√	√	√	Intergenic (−134/+158)	*CacPP4_08380* ←/← *CacPP4_08390*	hypothetical protein/Tm-1-like protein
1,007,908	C→A	√			R157S (CGT→AGT)	*sdhA_1* →	succinate dehydrogenase flavoprotein subunit
1,376,794	T→G	√	√	√	Intergenic (+755/+378)	*CacPP4_12500* →/← *ocd*	hypothetical protein/2,3-diaminopropionate biosynthesis protein SbnB
1,524,989	T→C	√	√	√	H37R (CAC→CGC)	*dprA* ←	putative DNA processing protein DprA
1,987,014	(C)_12→14_		√	√	intergenic (−71/+204)	*CacPP4_17970* ←/← *CacPP4_17980*	adhesin/choice-of-anchor M domain-containing protein
1,987,014	(C)_12→15_	√			Intergenic (−71/+204)		
2,115,863	T→C	√	√	√	G26G (GGT→GGC)	*oxyR* →	LysR family transcriptional regulator
2,173,362	G→C	√	√	√	S70W (TCG→TGG)	*CacPP4_19570* ←	ThuA domain-containing protein
2,488,946	A→C	√	√	√	Intergenic (−1723/+263)	*CacPP4_22410* ←/← *CacPP4_22420*	16S rRNA (guanine(527)-N(7))-methyltransferase/alanine racemase

√, Bacterial Isolate that Contains the gene. Synonymous mutations were presented in green, and non-synonymous mutations were in blue. Arrows in the Gene column indicate the transcription direction of the gene next to it, relative to the reference sequence (→: forward strand; ←: reverse strand).

## Data Availability

The phage KIT09 sequence was annotated and submitted to GenBank (accession number: PX403247; https://www.ncbi.nlm.nih.gov/nucleotide/, accessed on 23 September 2025).

## Supplementary Materials

The following supporting information can be downloaded at: https://www.mdpi.com/article/10.3390/ijms262412166/s1.

## Abbreviations

The following abbreviations are used in this manuscript:

NITE	National Institute of Technology and Evaluation
NBRC	NITE Biological Resources Center
CRISPR	Clustered Regularly Interspaced Short Palindromic Repeats
MOI	Multiplicity of Infection
SNP	Single-nucleotide polymorphism
