# Supplementary material for: Characterisation of the Novel Cutibacterium acnes Phage KIT09 and First Report of CRISPR-Cas-Independent Bacteriophage Resistance in Phylotype IA1
Source: Int J Mol Sci. 2025 Dec 18;26(24):12166. doi: 10.3390/ijms262412166 (PMC12733950; doi:10.3390/ijms262412166)
Supplement: Supplementary file 1 [file ijms-26-12166-s001.zip › ijms-4024250-supplementary.pdf]

Supplementary Materials

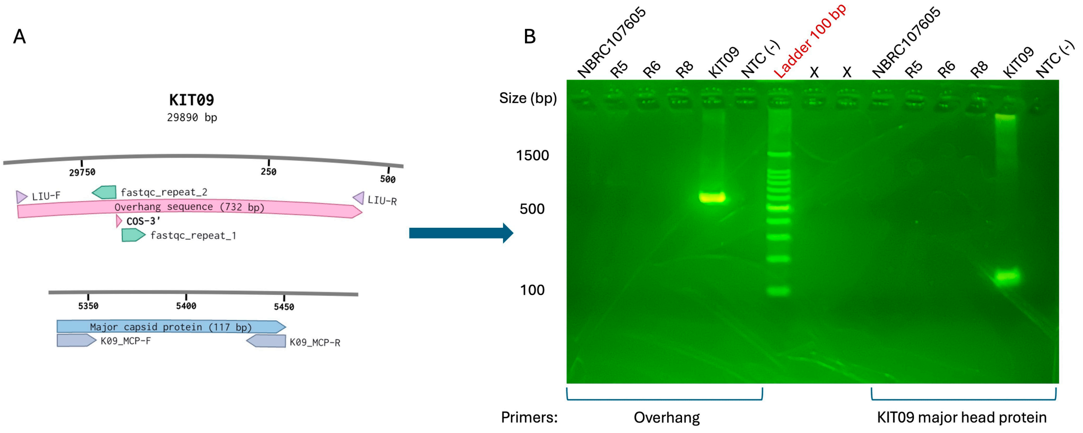

Figure S1. Illustration of the analysed overhang and major capsid protein sequence (**A**). Electrophoresis of PCR products targeting bridging fragments (overhangs) between the ends of KIT09's circular genome using the LIU primer (Liu et al., 2015) and the major head protein of KIT09 (K09_mcp) (**B**). Template DNA was extracted from the host strain (NBRC 107605) and the resistant isolates (R5, R6, and R8). The primers used are listed in Table S2. KIT09, KIT09 DNA was used as a positive control template; NTC(-), nuclease-free water was used as a negative control.

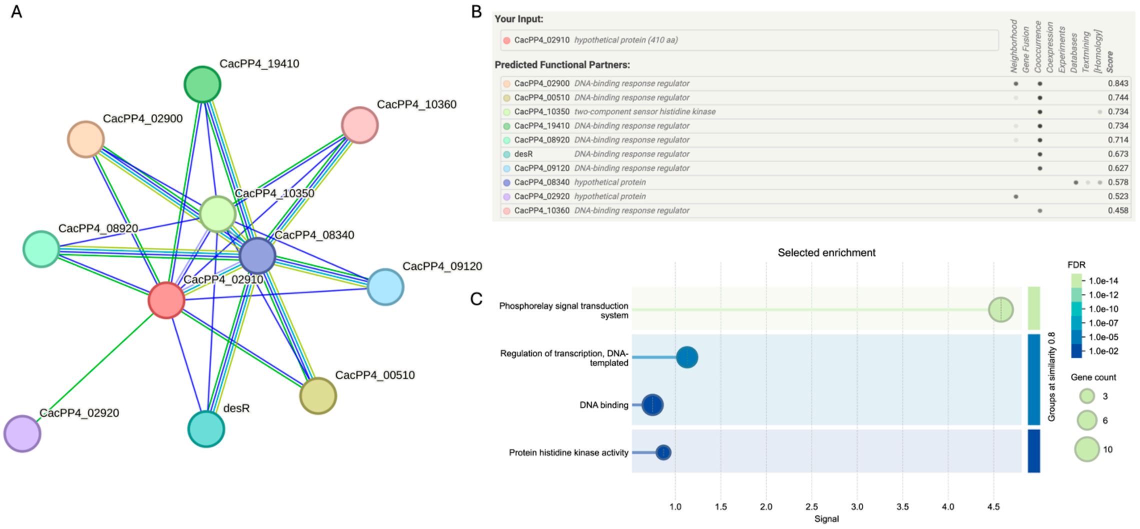

Figure S2. Protein-protein association network and functional enrichment analysis using STRING version 12.0. Protein-protein association network with CacPP4_02910 as the central node (**A**), with legends presented in (**B**). Functional enrichment analysis is presented in (**C**).

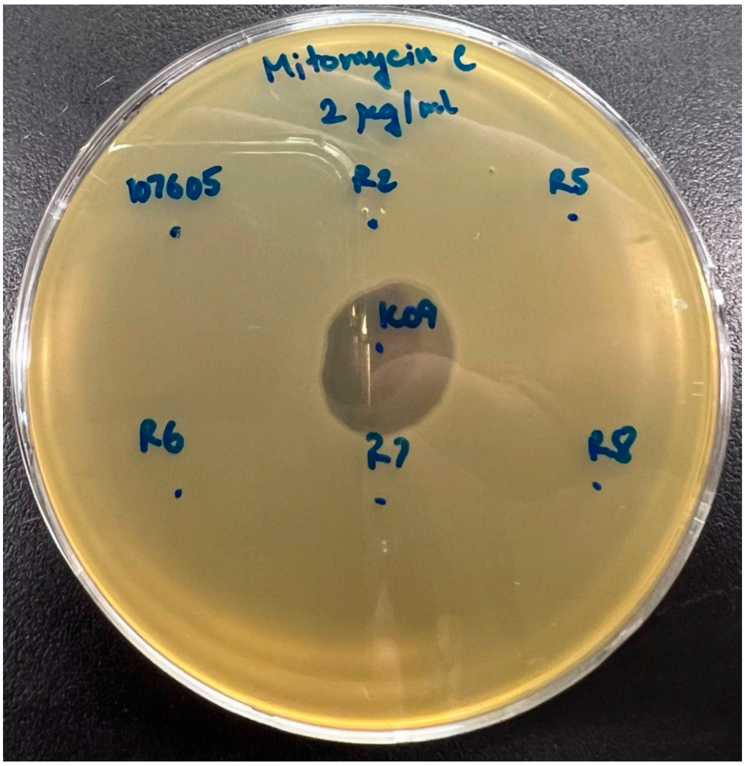

Figure S3. Prophage induction by adding Mitomycin C (2 μg/mL) to bacteria culture of the resistant isolates. The supernatant of the isolates and wild type after 24 h culture with Mitomycin C was spotted on the NBRC 107605 bacterial lawn to check for sign of prophages. KIT09 (K09) was also spotted to compare the bacteriolysis to the collected supernatants.

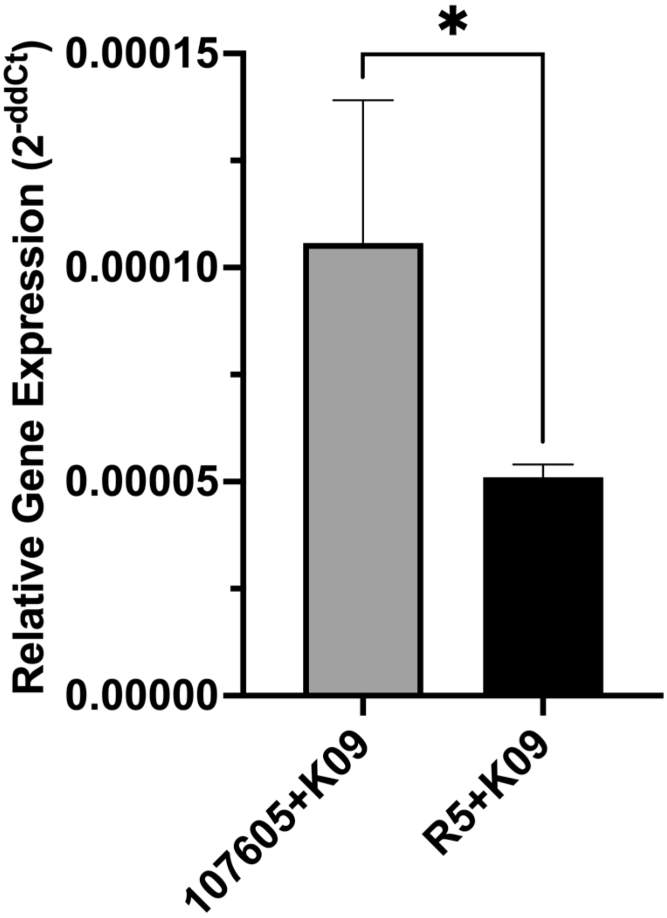

Figure S4. mRNA expression levels of phage KIT09 genes in *C. acnes* NBRC 107605 and R5 were measured 20 min post-infection. The primers used are listed in Table S2. The host strain (NBRC 107605) and R5 are shown in grey and black, respectively. MCP, phage gene encoding major capsid protein.

Table S1. CDS prediction and functional annotation of 42 CDSs from KIT09.

contig_id	cds_id	start	end	strand	phrog	function	product
**KIT09**	ACOH37_CDS_0001	1	312	+	1897	head and packaging	terminase small subunit
**KIT09**	ACOH37_CDS_0002	312	1823	+	9	head and packaging	terminase large subunit
**KIT09**	ACOH37_CDS_0003	1820	3145	+	104	head and packaging	portal protein
**KIT09**	ACOH37_CDS_0004	3149	3904	+	193	head and packaging	head maturation protease
**KIT09**	ACOH37_CDS_0005	4014	4571	+	1879	head and packaging	head scaffolding protein
**KIT09**	ACOH37_CDS_0006	4578	5525	+	10	head and packaging	major head protein
**KIT09**	ACOH37_CDS_0007	5569	6030	+	1290	connector	head-tail adaptor
**KIT09**	ACOH37_CDS_0008	6032	6379	+	501	connector	head closure Hc1
**KIT09**	ACOH37_CDS_0009	6385	6675	+	387	connector	neck protein
**KIT09**	ACOH37_CDS_0010	6672	7043	+	1892	tail	minor tail protein
**KIT09**	ACOH37_CDS_0011	7104	7736	+	117	tail	major tail protein
**KIT09**	ACOH37_CDS_0012	7764	8060	+	1927	tail	tail assembly chaperone
**KIT09**	ACOH37_CDS_0013	8159	8446	+	3166	unknown function	tail assembly chaperone
**KIT09**	ACOH37_CDS_0014	8454	11219	+	58	tail	tail length tape measure protein
**KIT09**	ACOH37_CDS_0015	11235	12182	+	106	tail	minor tail protein
**KIT09**	ACOH37_CDS_0016	12190	13347	+	110	tail	minor tail protein
**KIT09**	ACOH37_CDS_0017	13368	14186	+	1910	other	lectin
**KIT09**	ACOH37_CDS_0018	14485	15291	+	1806	tail	Collagen-like minor tail
**KIT09**	ACOH37_CDS_0019	15345	16205	+	670	lysis	endolysin
**KIT09**	ACOH37_CDS_0020	16218	16613	+	443	lysis	holin
**KIT09**	ACOH37_CDS_0021	17194	16703	-	2092	unknown function	hypothetical protein
**KIT09**	ACOH37_CDS_0022	17594	17199	-	3512	transcription regulation	sigma factor
**KIT09**	ACOH37_CDS_0023	17882	17598	-	1947	unknown function	hypothetical protein
**KIT09**	ACOH37_CDS_0024	18215	17895	-	1340	unknown function	hypothetical protein
**KIT09**	ACOH37_CDS_0025	19338	18673	-	284	DNA, RNA and nucleotide metabolism	exonuclease
**KIT09**	ACOH37_CDS_0026	19529	19335	-	1923	unknown function	hypothetical protein
**KIT09**	ACOH37_CDS_0027	20073	19513	-	2548	unknown function	hypothetical protein
**KIT09**	ACOH37_CDS_0028	20636	20070	-	1881	unknown function	hypothetical protein
**KIT09**	ACOH37_CDS_0029	21352	20681	-	849	DNA, RNA and nucleotide metabolism	DNA primase
**KIT09**	ACOH37_CDS_0030	21387	21575	+	No_PHROG	unknown function	hypothetical protein
**KIT09**	ACOH37_CDS_0031	21906	21550	-	244	DNA, RNA and nucleotide metabolism	RusA-like Holliday junction resolvase
**KIT09**	ACOH37_CDS_0032	22769	21906	-	243	DNA, RNA and nucleotide metabolism	DnaB-like replicative helicase
**KIT09**	ACOH37_CDS_0033	23274	22810	-	6015	DNA, RNA and nucleotide metabolism	single-strand DNA-binding protein
**KIT09**	ACOH37_CDS_0034	23733	23323	-	1890	unknown function	hypothetical protein
**KIT09**	ACOH37_CDS_0035	24671	23730	-	1870	DNA, RNA and nucleotide metabolism	exonuclease
**KIT09**	ACOH37_CDS_0036	25024	24671	-	801	DNA, RNA and nucleotide metabolism	dATP / dGTP pyrophosphohydrolase
**KIT09**	ACOH37_CDS_0037	25334	25131	-	1937	unknown function	hypothetical protein
**KIT09**	ACOH37_CDS_0038	25558	25331	-	1884	unknown function	hypothetical protein
**KIT09**	ACOH37_CDS_0039	26108	25575	-	1908	unknown function	hypothetical protein
**KIT09**	ACOH37_CDS_0040	26476	26192	-	7707	unknown function	hypothetical protein
**KIT09**	ACOH37_CDS_0041	26915	26604	-	919	unknown function	hypothetical protein
**KIT09**	ACOH37_CDS_0042	27230	26955	-	919	unknown function	hypothetical protein

Table S2. Primers used for PCR and qPCR.

Gene name	Primer Name	Primer F/R (5′-3′)
K09_MCP	K09_MCP-F	TTC CAG CGT AAC TTC CCG AT
	K09_MCP-R	AGC CAC ATA CAG AAC AGC CT
NBRC 107605 16S	16S-F	GAA AGG CCC TGC TTT TGT GG
	16S-R	TGA TAA GCC GCG AGT CCA TC
KIT09 overhang	LIU-F	CCG AAG CCG ACC ACA TCA CAC
	LIU-R	TCA TCC AAC AAC TGC TGC TGC C
